# Psychiatry

**Published:** 1906-01

**Authors:** 


**Psychiatry.** A Textbook for Students and Practitioners. By **Stewart Paton,** M.D., Associate in Psychiatry, the John Hopkins University, Baltimore, Director of the Laboratory of the Sheppard and Enoch Pratt Hospital, Towson, Md. Octavo, pp. 618. Illustrated. Philadelphia and London: J. B. Lippincott Co. 1905.

This author does not present an exhaustive treatise of psychiatry, but has selected that aspect of the subject which is in accordance with the results of observation at the bedside and in the laboratory. After discussing the importance, methods, and scope of psychiatry, as well as the relation of the diseased processes to pathological changes, he takes up the symptoms of the various forms of insanity as found in the insane. He discusses quite fully the anomaly of conduct with special reference to the so-called moral insanity. Although he does not place himself clearly on record, we come to the conclusion that the author does not believe in moral insanity without the accompanying intellectual defects.

In his chapter on the methods of the examination of patients, Paton gives full directions for the investigation of personal history and the noting of the physical and mental systems of the patients at the time of the examination.

That which will strike the majority of readers as new is the examination of the cerebrospinal fluid. The technic recommended by Sicard is the withdrawal of three or four centimeters of the fluid into a sterile tube; after centrifugation, the fluid is poured into a re-agent glass and tested for lymphocytes. Lymphocytosis of varying degrees has been noted in dementia, paralytica, tabes, tuberculosis, and in meningitis in which there is an involvment of the meninges. This procedure is indicated when it is necessary to differentiate between functional and organic disorders.

In the chapter on the treatment of insanity we note with pleasure, the strong position Paton takes in condemning the practice of allowing sallow-faced patients to remain in doors for hours at a time, when they might be out of doors and kept occupied by massage and gymnastics. He recommends that certain of the attendants should be skilled in massage, and in giving instructions to patients in various gymnastic exercise. With this view we are heartily in accord. Of medicinal theraputic measures we do not note anything unusual in the use of drugs.

In considering the causes of insanity, the author calls attention to the fact that there is no greater fallacy than the education given in our public schools as the cure-all for the many deficiencies of our social and political system. He believes that the enormous increase of nervous and mental diseases is the immediate result of trying to educate individuals unable to withstand the strain imposed upon them. It is a curious comment, he says, that so little effort is made along the lines of preventing alienation, while the right is given to prevent the spread of measles and scarlet fever. What form of education is best adapted among children, and to what degree of mental activity is woman capable of without impairing her physical vigor? This question he says, calls for the urgent consideration of those who are familiar with the methods of investigation of the difficulties connected with the functional activity of the central nervous system.

In his study of the classification of insanity, we note with regret the author's departure from a discussion of the disease group we are accustomed to call melancholia. We consider this a mistake; that melancholia is a disease and has been too well known and too often recognised to warrant its being classed in the manic-depressive group. We believe it is unwise to use the term manic-depressive insanity to describe those cases which never show any signs of mania. We agree entirely with Dana when he states that he prefers to use the term manic-depressive insanity only when there is really a combination of the two.

The author's style in many places is made difficult for the reader who is not accustomed to the use of new words recently introduced in psychiatry. For example, Paton uses such words as Acathisia, Akoasmata, Algolagnia, Topoalgia and Embolophrasia. The subject is difficult enough without loading the literature with these phrases imported from foreign languages. In addition to these words to which attention is called, we object decidedly to the author's assumption of inventing new words in the English language. Perseveration is of the author's coinage and is defined by him as "perseverance in an act." As we do not find the word in the Century Dictionary and we have never felt the need of it, we regret a style which keeps the reader guessing as to what is really intended. This is especially annoying when the author is a capable observer, as is the case in the present instance.

In his discussion of the epileptic group of insanity we note, with interest, that Paton is firm in his belief that bromides are generally used in the prevention of excitement, though we regret, that he has neglected to state in what doses and for how long he gives the bromides, as this is at present a much mooted question.

The book itself is a welcome addition to the literature of psychiatry; it is fully abreast of the period and presents the views of many of the leading teachers and students in Europe. Our great regret is that the author has not included a chapter on melancholia, and that he has frequently clothed his ideas in a language which increases the difficulty of the subject.

